# Individual body mass and length dataset for over 12,000 fish from Iberian streams

**DOI:** 10.1016/j.dib.2022.108248

**Published:** 2022-05-06

**Authors:** Ignasi Arranz, Sandra Brucet, Mireia Bartrons, Carmen García-Comas, Carles Alcaraz, Mònica Bardina, Patricia Navarro Barquero, Frederic Casals, Nuno Caiola, María Concepción Duran, Emili García-Berthou, Alberto Maceda-Veiga, Antoni Munné, María José Rodríguez-Pérez, Carolina Solà, Adolfo de Sostoa, Lluís Benejam

**Affiliations:** a Aquatic Ecology Group, University of Vic - Central University of Catalonia, Vic, Catalonia, Spain; b Laboratoire Évolution et Diversité Biologique, Université de Toulouse, CNRS, IRD, UPS, 118 route de Narbonne, Toulouse, France; c Catalan Institution for Research and Advanced Studies (ICREA), Barcelona, Spain; d Department of Marine Biology and Oceanography, Institut de Ciències del Mar, ICM-CSIC, Barcelona, Spain; e IRTA - Institute of Agrifood Research and Technology, Marine and Continental Waters Program, La Ràpita, Catalonia, Spain; f Agència Catalana de l'Aigua, C/Provença 260, Barcelona, Spain; g Confederación Hidrográfica del Ebro Paseo Sagasta, Zaragoza, Spain; h Department of Animal Science, Lleida University, Campus ETSEA – Edifici 1, Av. Alcalde Rovira Roure 191, Lleida, Catalonia, Spain; i Forest Science and Technology Centre of Catalonia-CTFC, Ctra. De St. Llorenç de Morunys, km 2, Solsona, Catalonia, Spain; j Climate Resilience Center (CRC), Amposta, Spain; k Department of Climate Change, EURECAT, Amposta, Spain; l GRECO, Institute of Aquatic Ecology, University of Girona, Girona, Catalonia, Spain; m Integrative Zoology Lab, Departament de Biologia Evolutiva, Ecologia i Ciències Ambientals, Institut de Recerca de la Biodiversitat, Universitat de Barcelona (UB), 08028 Barcelona, Spain

**Keywords:** body size, freshwater fish, functional traits, intraspecific variability, environmental variables, human-altered ecosystems, Mediterranean streams, water quality

## Abstract

We provide a unique fish individual body size dataset collected from our own sampling and public sources in north-eastern Spain. The dataset includes individual body size measures (fork length and mass) of 12,288 individuals of 24 fish species within 10 families collected at 118 locations in large rivers and small streams. Fish were caught by one-pass electrofishing following European standard protocols. The fish dataset has information on the local instream conditions including climatic variables (i.e., temperature and precipitation), topography (i.e., altitude), nutrient concentration (i.e., total phosphorus and nitrates), and the IMPRESS values (a measure of cumulative human impacts in lotic ecosystems). The potential uses of this new fish dataset are manifold, including developing size-based indices to further estimate the ecological status of freshwater ecosystems, allometric models, and analysis of variation in body size structure along environmental gradients.

## Specifications Table

**Table:** 

Subject	Biology, Zoology
Specific subject area	Freshwater fish ecology, community ecology, environmental science
Type of data	Table
How data were acquired	All data were provided by two public institutions: *Agència Catalana de l'Aigua* (ACA) and *Confederación Hidrográfica del Ebro* (CHE). Fish individual body sizes, water samples, anthropogenic pressures, and topographic variables were obtained in the field at each stream location following official biomonitoring programs. Field data was noted in sheets and later organized in files in text format. Water samples were analysed in the laboratory to obtain nutrient concentrations. The climate data was retrieved from the Global Climate Data. All data were curated and analysed in the statistical environment R 4.1.0 (R Development Core Team, 2021) before making them available here in three files in text format with tab-separated values.
Data format	Raw
Parameters for data collection	Length (mm) and mass (g) of 12,288 specimens belonging to 24 fish species is provided. Additionally, geographic coordinates, sampling date, sampling area, mean annual nutrient concentration (i.e., total phosphorus, total nitrogen), topography (i.e., altitude), climate-related variables (i.e., temperature, precipitation) and IMPRESS values are also given for the 118 stream locations.
Description of data collection	Fish were captured by one-pass electrofishing sampling during May and October between 2003 and 2009 in 118 stream locations of the NE Iberian Peninsula. The fish caught were counted, measured, and identified to the species level. The fish sampling area was recorded for each sampling. For each location, stream nutrient concentrations were quantified through water analyses in the laboratory. Climate-related variables and topography were estimated from the WorldClim database. IMPRESS values, a measure of the cumulative pressures in Mediterranean streams, were estimated following standardized protocols.
Data source location	Data covers a latitudinal gradient between 40.73 and 43.08°N, and a longitudinal gradient between 4.17°W and 2.29°E in the north-eastern part of the Iberian Peninsula. Stream locations are mainly in the Ebro basin (*n* = 103) but a few of them belong to the smaller river basins of Llobregat (*n* = 6), Besós (*n* = 5), Francolí (*n* = 3), and Gaià (*n* = 1).
Data accessibility	Repository name: Zenodo Data identification number: doi.org/10.5281/zenodo.6327188 Direct link to the dataset: https://zenodo.org/record/6327188#.YiHyKxOZOw4
Related research articles	Arranz, S. Brucet, M. Bartrons, C. García-Comas, L. Benejam, Fish size spectra are affected by nutrient concentration and relative abundance of non-native species across streams of the NE Iberian Peninsula, Sci. Total Environ. 795 (2021) 148792. https://doi.org/10.1016/j.scitotenv.2021.148792.

## Value of the Data

• Assessing biodiversity status and trends in fish communities is critical to maintaining ecosystem services.
• Individual body-size fish data is rarely available but size-based approaches can be useful to integrate with official biomonitoring programs.
• The database can be used by other researchers to investigate the community patterns in Mediterranean streams and to assess the biological status of fish species.
• The database also contributes to enhancing the knowledge of the ecology and biology of fish in the streams of the Iberian region, a region heavily impacted by human activities but holding a unique fauna.

## 1 Data Description

**Fig. 1:**
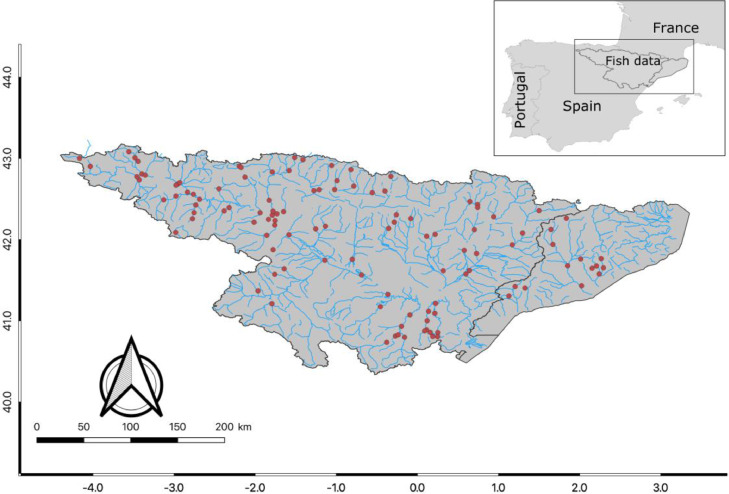
Map with the geographical distribution of the stream sampling sites. Map scale refers to the Ebro basin and internal basins of Catalonia with the hydrological network and stream sampling sites in red.

The present data article includes 12,288 of individual body length (mm) and body weight (g) of 24 fish species in 118 stream locations in the north-eastern Iberian Peninsula (latitudinal gradient from 40.73° to 43.08°N and longitudinal gradient from 4.17°W to 2.29°E; Fig. 1). Specifically, stream locations are in an area mostly characterized by the Mediterranean climate and located within the west part of the Palearctic ecoregion, within the Ebro basin (*n* = 103) and smaller river basins of Llobregat (*n* = 6), Besós (*n* = 5), Francolí (*n* = 3), and Gaià (*n* = 1). The accessibility of this data came from a recent scientific publication by Arranz et al. [1]. The data was collected from two main public institutions named *Agència Catalana de l'Aigua* (hereafter, ACA) and *Confederación Hidrográfica del Ebro* (hereafter, CHE). The dataset can be found in the Zenodo data repository [2] and includes three text files with tab-separated values. The first file is named *0_Data_Dictionary* and contains a detailed description of the variables in the following two files including the definition, and attribute of each variable (see also Table 1 for a summary). The second file is named *1_stream_information* and provides the complete records of the stream locations (toponomy and data source from the two public institutions), fish sampling (date, number of fish caught and sampling area), local environmental information, and a measure of anthropogenic pressure of each stream location. Most of the environmental information, which includes water samples and topographic variables, was obtained on the same day of the fish sampling. Water samples were frozen and transported to the research laboratory for the analysis of nutrient concentrations (see Material and Methods for further details). Additional environmental data, which includes climate information, was obtained from the geographic coordinates of each location (longitudinal and latitudinal in a World Geodetic System 84, WGS84) and retrieved from the Global Climate Data (hereafter, WorldClim) with a spatial resolution of 1 km^2^ using the statistical environment R version 4.1.0 [3]. The anthropogenic pressure was described as the IMPRESS value, a standardized metric derived for the Water Framework Directive to assess the ecological health of European streams [4]. It evaluates multiple pressures including hydrological alterations, point and diffuse source of pollution, and riparian landscape changes [4]. The third and the last file, named *2_fish_information*, contains the individual fish body size (length and weight) and the scientific Latin name of the fish species. The files *1_stream_information* and *2_fish_information* can be concatenated according to the variable in both files named *Code_ID*, which consists of a numeric sequence of values from 1 to 118. In addition, we provide sensitivity analyses of the fish individual body size through mass-length relationships for each fish species. The mass-length relationships can be useful for other studies in the same region when the direct measures of individual fish body mass, usually more time-consuming to measure in the field, are not available. All data were curated, organized, and analysed in the statistical environment R version 4.1.0 [3].

**Table 1:** Information of the variables describing the new fish body size data in Iberian streams.

Variable name	Description	Variable type	File number
Code_ID	Unique number identifying a stream location	Categorical	1,2
Stream	Name of the stream	String	1,2
Location	Name of the location	String	1,2
Lat_degree	Latitudinal coordinates in WGS 84	Numeric	1
Long_degree	Longitudinal coordinates in WGS 84	Numeric	1
Source	Public source where the fish data was obtained	String	1
Day	Day of the fish sampling	Numeric	1
Month	Month of the fish sampling	Numeric	1
Year	Year of the fish sampling	Numeric	1
Fish_number	Total number of fish caught in each stream location	Numeric	1
SA_m2	Sampling area (m^2^)	Numeric	1
TP_mg.l	Mean annual concentration of total phosphorus (mg•l^−1^)	Numeric	1
TN_mg.l	Mean annual concentration of total nitrates (mg•l^−1^)	Numeric	1
Temp_degree_celcius	Mean annual air temperature (°C)	Numeric	1
Prec_mm	Mean annual precipitation (mm)	Numeric	1
Alt_m	Altitude (m a.s.l.)	Numeric	1
IMPRESS	Cumulative pressures (unitless)	Numeric	1
Family	Family latin name	String	2
Genus	Genus latin name	String	2
Species	Species latin name	String	2
Scientific_name	Full scientific name of the fish species	String	2
Length_mm	Individual body length (mm)	Numeric	2
Mass_g	Individual body mass (g)	Numeric	2

## 2 Experimental Design, Materials and Methods

### 2.1 Data selection

Complete fish and stream data are available at https://www.chebro.es/ for the CHE and from the corresponding author upon reasonable request. We carried out initial data screening to select comparable stream locations with robust data that can largely represent the species composition, and body size structure of each fish assemblage among stream locations. To do this, we limited the selection of streams sampled from May to October (both included) to avoid the transient effects of seasonal events and sudden increases in fish density from spring reproduction. We further screened samples by a floor of 34 individuals measured in order to minimize statistical biases associated with fish low catches. The number of catches varied substantially among sites (median = 91; SD= 54.3). In cases of more than one sampling occasion per year within the seasonal range, we kept one sample (the more with more fishes caught). In total, the data selection comprehended 118 stream reaches sampled between 2003 and 2009.

### 2.2 Fish sampling

Fish sampling was carried out through one-pass electrofishing (from 2.5 to 4.5 kW and from 300 V to 800 V, pulsed DC current) with the help of operators holding dip nets to catch fish stunned by the electric field [5]. Each sampling location covered all mesohabitats (e.g., runs, riffles, pools) and sampling stream length varied according to the stream width (from 20 m in small streams to 50 m of river margin [5]). Depending on the stream width, electrofishing was carried out by boat (usually in near-shore areas) in large rivers or by wading foot in small streams [5]. The fish capturability of the electrofishing gears used has been analysed elsewhere, and comprises one of the most efficient methods for biomonitoring programs [6,7]. Water conductivity was measured prior to electrofishing to determine the appropriate output voltage for effective sampling but minimizing unwanted fish mortality. Additionally, fish sampling was done when the water temperature was > 5°C because fish catchability is low below that temperature [5]. Fishes stunned by electrofishing were anesthetized using MS222 (tricaine methanesulfonate), identified to species level, measured (fork length, mm), weighed (g), checked for DELT anomalies (Deformities, Eroded Fins, Lesions, and Tumors), and released back to the stream after recovery from MS222.

### 2.3 Local abiotic information

A one-liter water sample was taken in each stream location, and transported in cool boxes in the laboratory. Water samples were immediately filtered in the laboratory through Whatman GF/F filters (0.7 µm pore size and 47 mm diameter), and stored frozen until nutrient analyses, following the International Organization for Standardization (ISO). For the concentration of total phosphates (mg•l^−1^), a Continuous Flow Analysis (CFA) was conducted for each sample in each stream location following the UNE-EN ISO 6878:2005 [8]. For the total nitrates (mg•l^−1^), a chemiluminescent technique for the determination of nanomolar quantities of nitrate, nitrate plus nitrite, or nitrite alone in the stream water was conducted in each stream location following the UNE-EN ISO 11905-1:1998 [9]. Climate-related variables were represented by mean annual air temperature (°C), precipitation (mm), and altitude (m). They were calculated from the geographic coordinates of each location (that is, longitudinal and latitudinal in a World Geodetic System 84, WGS84) as the 20-year average at 1 km^2^ spatial resolution from the WorldClim database [10]. The calculations of the climate-related variables were carried out using the statistical environment R version 4.1.0 [3].

**Table 2:** Information of the mass-length relationships of the stream fish species with more than 25 individuals. Descriptive and regression statistics of the linear regressions (log_10_*M* = *a* + *b* log_10_*L*) between fish individual mass (*M*) and length (*L*) for each fish species. Length is fork length, except for eel, mosquitofish, blenny, and catfish, where it is total length. Min. = minimum, max. = maximum; CI = confidence interval.

Scientific name	Common name	Family	*n*	Min. length (mm)	Max. length (mm)	Min. mass (g)	Max. mass (g)	*a*	95% CI of *a*	*b*	95% CI of *b*	*r*^2^
*Achondrostoma arcasii*	Bermejuela	Leuciscidae	302	19	105	0.1	16	-5.494	(-5.634 – -5.353)	3.342	(3.261 – 3.423)	0.956
*Alburnus alburnus*	Bleak	Cyprinidae	387	23	191	0.1	100.7	-5.651	(-5.777 – -5.526)	3.318	(3.252 – 3.385)	0.961
*Anguilla anguilla*	Common eel	Anguillidae	28	220	700	14.3	925	-6.282	(-6.911 – -5.653)	3.218	(2.973 – 3.462)	0.964
*Barbatula quignardi*	Languedoc stone loach	Nemacheilidae	681	22	101	0.1	8.4	-5.356	(-5.434 – -5.278)	3.140	(3.096 – 3.184)	0.966
*Barbus haasi*	Catalonian barbel	Cyprinidae	1110	18	244	0.1	240	-5.141	(-5.183 – -5.099)	3.132	(3.110 – 3.153)	0.987
*Barbus meridionalis*	Mediterranean barbel	Cyprinidae	175	30	144	0.4	53.7	-4.964	(-5.034 – -4.893)	3.101	(3.063 – 3.139)	0.993
*Cobitis calderoni*	Loach	Cobitidae	193	30	85	0.1	1.9	-5.408	(-5.794 – -5.023)	2.985	(2.763 – 3.208)	0.784
*Cyprinus carpio*	Common carp	Cyprinidae	89	43	487	0.8	2512	-4.763	(-4.856 – -4.670)	3.022	(2.981 – 3.062)	0.996
*Gambusia holbrooki*	Eastern mosquitofish	Poeciliidae	69	20	85	0.1	8	-4.586	(-4.728 – -4.443)	2.750	(2.647 – 2.853)	0.977
*Gobio lozanoi*	Iberian gudgeon	Cyprinidae	1226	18	127	0.1	29.7	-5.344	(-5.421 – -5.267)	3.232	(3.190 – 3.274)	0.949
*Lepomis gibbosus*	Pumpkinseed	Centrarchidae	48	50	120	3	42	-5.099	(-5.536 – -4.662)	3.225	(3.002 – 3.448)	0.947
*Luciobarbus graellsii*	Ebro barbel	Cyprinidae	2008	15	530	0.1	2000	-4.869	(-4.898 – -4.841)	2.996	(2.982 – 3.009)	0.990
*Parachondrostoma miegii*	Ebro nase	Cyprinidae	1893	23	216	0.1	140.9	-5.252	(-5.290 – -5.214)	3.152	(3.132 – 3.172)	0.981
*Phoxinus phoxinus*	Minnow	Leuciscidae	1955	19	109	0.1	14.6	-5.445	(-5.498 – -5.391)	3.296	(3.265 – 3.327)	0.958
*Salaria fluviatilis*	Freshwater blenny	Blenniidae	198	36	120	0.5	20	-5.344	(-5.523 – -5.166)	3.198	(3.104 – 3.293)	0.957
*Salmo trutta*	Brown trout	Salmonidae	1456	31	486	0.2	1146	-4.984	(-5.015 – -4.952)	3.023	(3.008 – 3.038)	0.991
*Silurus glanis*	Wels catfish	Siluridae	27	65	862	2	4500	-5.121	(-5.224 – -5.017)	2.967	(2.924 – 3.010)	0.999
*Squalius laietanus*	Catalan chub	Cyprinidae	328	36	284	0.6	320	-5.336	(-5.412 – -5.260)	3.211	(3.173 – 3.249)	0.988

### 2.4 IMPRESS values

The cumulative effects of anthropogenic pressures are represented by the IMPRESS values. IMPRESS is an analysis to identify the pressures and to assess impacts, derived by the Water Framework Directive (WFD) to assess the ecological health of European lotic habitats [4]. Specifically, the IMPRESS values encompass cumulative pressures related to the presence of contaminants, hydromorphological alterations, and land-use changes (see details and formulas for each pressure in [11,^12^,13]). A greater value of IMPRESS means greater anthropogenic pressure, and thus failure in achieving the Directive's environmental objectives [4]. The European Commission, in the context of WFD, developed a protocol to explain how to calculate the IMPRESS value using data on the presence of contaminants, hydromorphological alterations, and land-use changes [4]. Then, each public agency implemented this protocol using data collected by themselves [11,^12^,13]. The IMPRESS values for our dataset were collected from these public agencies. CHE provided IMPRESS data of Ebro basin and its affluents whereas ACA facilitated IMPRESS data from Llobregat, Besós, Francolí, and Gaià streams.

### 2.5 Data validation

In order to confirm the robustness of our stream fish dataset, we used mass-length relationships (MLs) for each fish species whose abundances reached more than 25 individuals (in total, 18 out of 24 fish species). We regressed log_10_ fish mass with log_10_ fish length of the focal species, and used the coefficient of determination (*r*^2^) as a measure of goodness of fit (Table 2). As fish measures taken from the field may often cause errors in the fish body sizes [14], we removed individuals in which the residuals from the MLs at log-log scale were two times higher than the standard deviation (in total, 405 outliers representing 3.19 % of the 18 fish species selected for MLs).

## Declaration of Competing Interest

The authors declare that they have no known competing financial interests or personal relationships that have or could be perceived to have influenced the work reported in this article.

## Data Availability

Arranz.etal.fish.DiB.2022 (Original data) (Zenodo).
